# Endothelial dysfunction is a potential contributor to multiple organ failure and mortality in aged mice subjected to septic shock: preclinical studies in a murine model of cecal ligation and puncture

**DOI:** 10.1186/s13054-014-0511-3

**Published:** 2014-09-16

**Authors:** Ciro Coletta, Katalin Módis, Gábor Oláh, Attila Brunyánszki, Daniela S Herzig, Edward R Sherwood, Zoltán Ungvári, Csaba Szabo

**Affiliations:** Department of Anesthesiology, The University of Texas Medical Branch at Galveston, 601 Harborside Drive, Galveston, TX 77555 USA; Reynolds Oklahoma Center on Aging, Department of Geriatric Medicine, University of Oklahoma Health Sciences Center, 920 Stanton L Young Boulevard, Oklahoma, OK 73104 USA; Cardiovascular and Metabolic Research Unit, Department of Biology, Lakehead University, 955 Oliver Road, Thunder Bay, ON P7B 5E1 Canada; Department of Anesthesiology, Vanderbilt University Medical Center, Research Division, 1161 Medical Center Drive, Nashville, TN 37232 USA

## Abstract

**Introduction:**

The goal of the current study was to investigate the effect of aging on the development of endothelial dysfunction in a murine model of sepsis, and to compare it with the effect of genetic deficiency of the endothelial isoform of nitric oxide synthase (eNOS).

**Methods:**

Cecal ligation and puncture (CLP) was used to induce sepsis in mice. Survival rates were monitored and plasma indices of organ function were measured. *Ex vivo* studies included the measurement of vascular function in thoracic aortic rings, assessment of oxidative stress/cellular injury in various organs and the measurement of mitochondrial function in isolated liver mitochondria.

**Results:**

eNOS deficiency and aging both exacerbated the mortality of sepsis. Both eNOS-deficient and aged mice exhibited a higher degree of sepsis-associated multiple organ dysfunction syndrome (MODS), infiltration of tissues with mononuclear cells and oxidative stress. A high degree of sepsis-induced vascular oxidative damage and endothelial dysfunction (evidenced by functional assays and multiple plasma markers of endothelial dysfunction) was detected in aortae isolated from both eNOS^−/−^ and aged mice. There was a significant worsening of sepsis-induced mitochondrial dysfunction, both in eNOS-deficient mice and in aged mice. Comparison of the surviving and non-surviving groups of animals indicated that the severity of endothelial dysfunction may be a predictor of mortality of mice subjected to CLP-induced sepsis.

**Conclusions:**

Based on the studies in eNOS mice, we conclude that the lack of endothelial nitric oxide production, on its own, may be sufficient to markedly exacerbate the severity of septic shock. Aging markedly worsens the degree of endothelial dysfunction in sepsis, yielding a significant worsening of the overall outcome. Thus, endothelial dysfunction may constitute an early predictor and independent contributor to sepsis-associated MODS and mortality in aged mice.

## Introduction

Sepsis, a life-threatening systemic inflammatory disease affects nearly 700,000 people annually in the United States. Severe sepsis occurs in one quarter of all intensive care unit (ICU) admissions and accounts for up to half of ICU bed days. Sepsis is associated with fatality rates of 20 to 40%. Sepsis-related health care costs amount to $17 billion/year in the United States alone. The frequency of sepsis is increasing by >5% per year, in excess of the growth and aging of our population. The outcome of sepsis is dramatically worse in older people: age alone is an independent predictor of mortality in sepsis [1,2].

Although the development of endothelial dysfunction is well established in various forms of shock, both in preclinical models [3–9] and in patients [10–14], to our knowledge, it has not been previously investigated whether endothelial dysfunction associated with aging worsens the outcome and increases mortality rates of septic shock. Thus, the purpose of the current study was to investigate, in a murine model of cecal ligation and puncture-induced septic shock, whether aging increases the severity of endothelial dysfunction, leading to a worsening of oxidative stress, tissue inflammation and, ultimately, multiple organ dysfunction syndrome (MODS) and death. Our experimental approach included a comparison of the effect of aging on the outcome of sepsis to the effect of complete deficiency of endothelial nitric oxide synthase (eNOS), in order to start exploring the possibility that endothelial dysfunction may be a potential contributor to mortality and MODS in aging animals subjected to sepsis.

## Materials and methods

### Animals

Male mice homozygous for the Nos3^tm1Unc^ targeted mutation (eNOS^−/−^) on a C57BL6 background were obtained from The Jackson Laboratory, Bar Harbor, ME, USA (strain name: B6.129P2-Nos3^tm1Unc^/J; stock number: 002684). Male C57BL/6 mice (six to eight weeks of age) were used as wild-type controls (Jackson stock number: 000664). Aged (24 months) C57BL/6 mice were obtained from colonies of the National Institute on Aging. Animals were kept in a 12 h/12 h light/dark cycle at 21 to 23°C with free access to a standard chow diet.

### Cecal ligation and puncture (CLP)

Acute sepsis was induced in mice by cecal ligation and puncture (CLP) as previously described [15] with modifications [16]. Briefly, mice were anesthetized by ketamine/xylazine cocktail (intraperitoneal (i.p.)), the abdomen was shaved, wiped with 70% isopropanol and a midline abdominal incision (1 to 2 cm) was performed. The cecum was exteriorized, ligated with a sterile silk suture 1 cm from the tip and double punctured with a 20-gauge needle. The cecum was squeezed to assure expression of a small amount of fecal material and was returned to the abdominal cavity. The incision was closed with auto-clips and kept clean by povidone-iodine (Betadine). Mice were resuscitated with i.p. injection of 1 ml of lactated Ringer’s solution. Sham-operated mice were treated as described above with the exception for the ligation and puncture of the cecum. Pain was prevented by buprenorphine (0.1 mg/kg; subcutaneous (s.c.) 30 minutes before surgery and every 12 hours thereafter). For the survival study, mice were constantly monitored for 48 hours. Mice that survived this period of time were euthanized by cervical dislocation. In a separate set of studies, mice were sacrificed 12 hours following CLP by opening the chest cavity with an incision in the diaphragm. A sample of whole blood was collected for analysis of organ function using a comprehensive metabolic panel. Aortae were isolated and assessed for endothelial function. Finally, major organs were snap frozen and kept at −80°C until use. All animal procedures described in this study have been approved by the University of Texas Medical Branch Institutional Animal Care and Use Committee.

### Complete metabolic panel

Samples of whole blood were collected from septic mice, placed in lithium-heparin tubes and immediately processed for quantitative determinations of alanine aminotransferase (ALT), albumin (ALB), alkaline phosphatase (ALP), amylase (AMY) total calcium (Ca^2+^), creatinine (CRE), glucose (GLU), phosphorus (PHOS), potassium (K^+^), sodium (Na^+^), total bilirubin (TBIL), total protein (TP), and urea nitrogen (BUN) as described [16] by a VetScan Chemistry Analyzer (Abaxis, Union City, CA, USA) using the VetScan Comprehensive Diagnostic Profile reagent rotor.

### Detection of plasma markers of cardiovascular disease (CVD panel)

Blood from CLP or sham-operated mice was collected in K_2_EDTA blood collection tubes and centrifuged at 4°C for 15 minutes at 2,000xg within 30 minutes of collection. Plasma was isolated, aliquoted and stored at −80°C until use. The EMD Millipore’s MILLIPLEX™ MAP Mouse CVD Magnetic Bead Panel 1 kit was used for the simultaneous quantification of the following analytes: sE-Selectin, sICAM-1, Pecam-1, sP-Selectin, PAI-1 total, proMMP-9, and thrombomodulin (Merck Millipore, Darmstadt, Germany). Luminex uses proprietary techniques to internally color code microspheres with two fluorescent dyes and to create distinctly colored bead sets of 500 5.6 μm polystyrene microspheres or 80 6.45 μm magnetic microspheres, each of which is coated with a specific capture antibody. After an analyte from a test sample is captured by the bead, a biotinylated detection antibody is introduced. The reaction mixture is then incubated with streptavidin-phycoerythrin (PE) conjugate, the reporter molecule, to complete the reaction on the surface of each microsphere. The Luminex instrument acquires and analyze data using the Luminex xMAP fluorescent detection method and the Luminex xPONENT™ acquisition software (Thermo Fisher Scientific, Waltham, MA, USA).

### Isolated mouse aortic ring assay

Thoracic aortae were isolated from mice 12 hours following CLP or sham procedures. Aortic rings (approximately 2 mm in length) were placed in 5 ml organ baths filled with oxygenated (95% O_2_ to 5% CO_2_) Krebs-Henseleit solution at 37°C, were mounted onto isometric force transducers (Kent Scientific, Minneapolis, MN, USA), and connected to a PowerLab data acquisition system (AD Instruments, Dunedin, New Zealand) for the detection of isometric tension, as described [17]. Aortic rings were allowed to equilibrate for 45 minutes under a resting tension of 1.5 g and subsequently contracted with phenylephrine (PE; 1 μM). Once a plateau was reached, cumulative concentration response curves to acetylcholine (Ach) or to the nitric oxide (NO) donor sodium nitroprusside (SNP) were performed in order to assess the smooth muscle relaxation in response to endothelium-derived or exogenously applied NO. The experiments were repeated in at least four aortic rings, each from a different mouse.

### Detection of protein oxidation by the OxyBlot assay

Immunodetection of protein carbonyl groups (index of oxidative stress) was performed in tissue homogenates from thoracic aortas collected from mice undergoing 12 hours of severe sepsis, using the OxyBlot kit (Millipore) as described [18]. Protein carbonyl groups were derivatized with 2, 4-dinitrophenylhydrazine (DNPH) generating a stable dinitrophenylhydrazone (DNP) product immunodetected by specific anti-DNP antibodies. Following derivatization of the aortic tissue homogenates with DNPH, 20 μg of protein extracts were subjected to SDS-polyacrylamide gel electrophoresis (SDS-PAGE) using NuPAGE Novex 4% to 12% Bis-Tris Midi gels (Invitrogen, Thermo Fisher Scientific, Carlsbad, CA, USA). After electrophoresis, the proteins were transferred to a PVDF membrane. Membranes were then blocked in Starting Block T20 (TBS) blocking buffer (Thermo Fisher Scientific) and incubated with a rabbit anti-DNP primary antibody (1:500) provided by the manufacturer. The primary antibody was detected using an OxyBlot supplied horseradish peroxidase (HRP)-conjugated anti-rabbit immunoglobulin (Ig)G secondary antibody. Immunoreactivity was visualized by chemiluminescent detection. The Oxyblot method produces a smear-like blot, indicative of the oxidative modification of multiple proteins of different molecular weights. Blots were scanned and a densitometric analysis was performed on the totality of the modified proteins using NIH Image J software.

### Determination of tissue lipid peroxidation: malondialdehyde assay

Tissue malondialdehyde (MDA) levels, an index of cellular injury/oxidative stress, were detected as described [16] in aorta, liver and mesenteric vascular bed homogenates from mice subjected to 12 hours of CLP-induced sepsis, using a fluorimetric MDA-specific lipid peroxidation assay kit (Enzo Life Sciences, Farmingdale, NY, USA) according to the manufacturer’s instructions. The assay is based on the BML-AK171 method in which two molecules of the chromogenic reagent N-methyl-2-phenylindole (NMPI) react with one molecule of MDA at 45°C to yield a stable carbocyanine dye with a maximum absorption at 586 nm.

### Myeloperoxidase activity assay

Myeloperoxidase activity was measured in aorta, liver and mesenteric vascular bed extracts from wild-type, eNOS^−/−^ and aged mice subjected to 12 hours of CLP-induced severe intra-abdominal sepsis, as described [16] using a commercially available myeloperoxidase (MPO) fluorometric detection kit (Enzo Life Sciences). The assay utilizes a non-fluorescent detection reagent, which is oxidized in the presence of hydrogen peroxide and MPO to produce its fluorescent analog. The fluorescence is measured at excitation wavelength of 530 to 571 nm and emission wavelength of 590 to 600 nm.

### Isolation of liver mitochondria

The IV lateral liver lobes were harvested from mice subjected to sham operation or CLP at 12 hours. Mitochondria from livers were isolated by differential centrifugation as described [19]. In brief, livers were homogenized on ice-cold mitochondrial isolation buffer (MSHE-BSA, pH 7.2) composed of 210 mM mannitol, 70 mM sucrose, 5 mM HEPES, 1 mM EGTA, and 0.5% (w/v) fatty acid-free BSA using a drill-driven teflon glass homogenizer. Homogenates were centrifuged at 600 g for 10 minutes at (4°C). Following centrifugation, the lipid phase was carefully removed by aspiration and the remaining supernatant was decanted to a separate tube and centrifuged at 10,000 g for 10 minutes (4°C). The supernatant fraction was removed, the pellet was suspended in MSHE-BSA, and the centrifugation procedure was repeated two more times. Finally, the pellet was resuspended in a small volume (approximately 100 μl) of MSHE-BSA. Total protein was determined using the Pierce BCA Protein Assay Reagent (Thermo Fisher Scientific). Mitochondrial preparations were used for bioenergetic analysis within 1 to 3 hours.

### Bioenergetic analysis in isolated mitochondria

The XF24 Extracellular Flux Analyzer (Seahorse Biosciences, North Billerica, MA, USA) was used to measure mitochondrial bioenergetic function, as described [19]. Respiration by the mitochondria (10 μg/well) was sequentially measured in a coupled state with substrate present (basal respiration, State 2), followed by State 3 (phosphorylating respiration, in the presence of adenosine 5′-diphosphate sodium salt (ADP) and substrate), State 4 (non-phosphorylating or resting respiration) following conversion of ADP to adenosine triphosphate (ATP), State 4o, induced with the addition of oligomycin. Next, maximal uncoupler-stimulated respiration (State 3u) was detected by the administration of the uncoupling agent carbonylcyanide-4-trifluorometh-oxyphenylhydrazone (FCCP). At the end of the experiment the Complex III inhibitor, antimycin A was applied to completely shut down the mitochondrial respiration. This ‘coupling assay’ examines the degree of coupling between the electron transport chain (ETC.), and the oxidative phosphorylation (OXPHOS), and can distinguish between ETC. and OXPHOS with respect to mitochondrial function/dysfunction. In a second set of studies, electron flow experiments were conducted. This method allows the functional assessment of selected mitochondrial complexes together in the same time frame. Mitochondrial electron transport was stimulated by the addition of pyruvate/malate (10 mM/2 mM, respectively, in order to enable the activity of all complexes); with succinate (10 mM, in the presence of the Complex I inhibitor rotenone, 2 μM, in order to direct the electron flow exclusively through complexes II, III and IV) or with the artificial substrates ascorbate/TMPD (10 mM/100 μM, respectively, in the presence of the Complex III inhibitor antimycin at 4 μM, in order to selectively activate Complex IV) (3).

### Statistical analysis

Data are shown as mean ± SEM. One-way and two-way ANOVA with Bonferroni’s multiple comparison test were used to detect differences between groups. Statistical calculations were performed using Graphpad Prism 5 analysis software (GraphPad Software, La Jolla, CA, USA).

## Results

### eNOS deficiency and aging both exacerbate the mortality of sepsis

**Figure 1:**
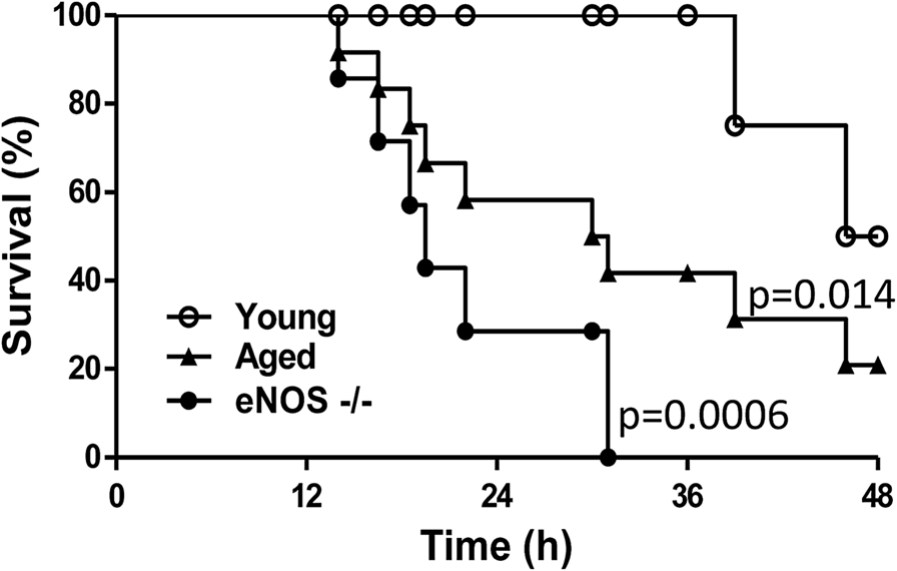
**Time course of survival rates in young, aged and eNOS** ^**−/−**^ **mice subjected to CLP.** Mice were subjected to acute sepsis by CLP as described in Methods and constantly monitored for 48 hours. Death events were annotated and the Kaplan-Meier estimate was used to compute the survival rates over time. Please notice that by 24 hours the eNOS-deficient mice showed the highest mortality rate (about 70%). At this time there was 40% mortality in the aged mice while no mortality in the wild-type control group (young mice). By 24 to 36 hours all the eNOS^−/−^ mice died, the mortality of the aged mice increased to about 60% while all the young mice survived. By 48 hours, the survival rates of the aged mice and the young mice were 20% and 50% respectively. Sham-operated mice showed no mortality in the 48-hour time frame (not shown) (*P* = 0.008 eNOS^−/−^ vs. young and *P* = 0.024 aged vs. young; n = 12/group). CLP, cecal ligation and puncture; eNOS, endothelial isoform of nitric oxide synthase.

To determine whether mice exhibited an eNOS deficiency- and/or an age-associated vulnerability to sepsis, we compared mortality rates of eNOS^−/−^ and aged (24 months) versus young mice (C57BL/6, approximately two months of age) in response to CLP. Mortality was assessed every 12 hours for two days. At the end of the second day, the mice that survived were sacrificed by opening the chest cavity under deep anesthesia. In our model of sepsis, approximately 70% of the eNOS deficient mice died by 24 hours, showing the highest mortality rate among the animal groups studied (Figure 1). At the same time, there was 40% mortality in the aged mice. In contrast, no mortality was detected in the wild-type control group. By 48 hours, the survival rates of the aged mice and the young were 20% and 50% respectively; none of the eNOS^−/−^ mice used in the study survived to 48 hours.

### eNOS deficiency and aging both induce a worsening of sepsis associated-multiple organ dysfunction (MOD), tissue inflammation and oxidative stress

**Figure 2:**
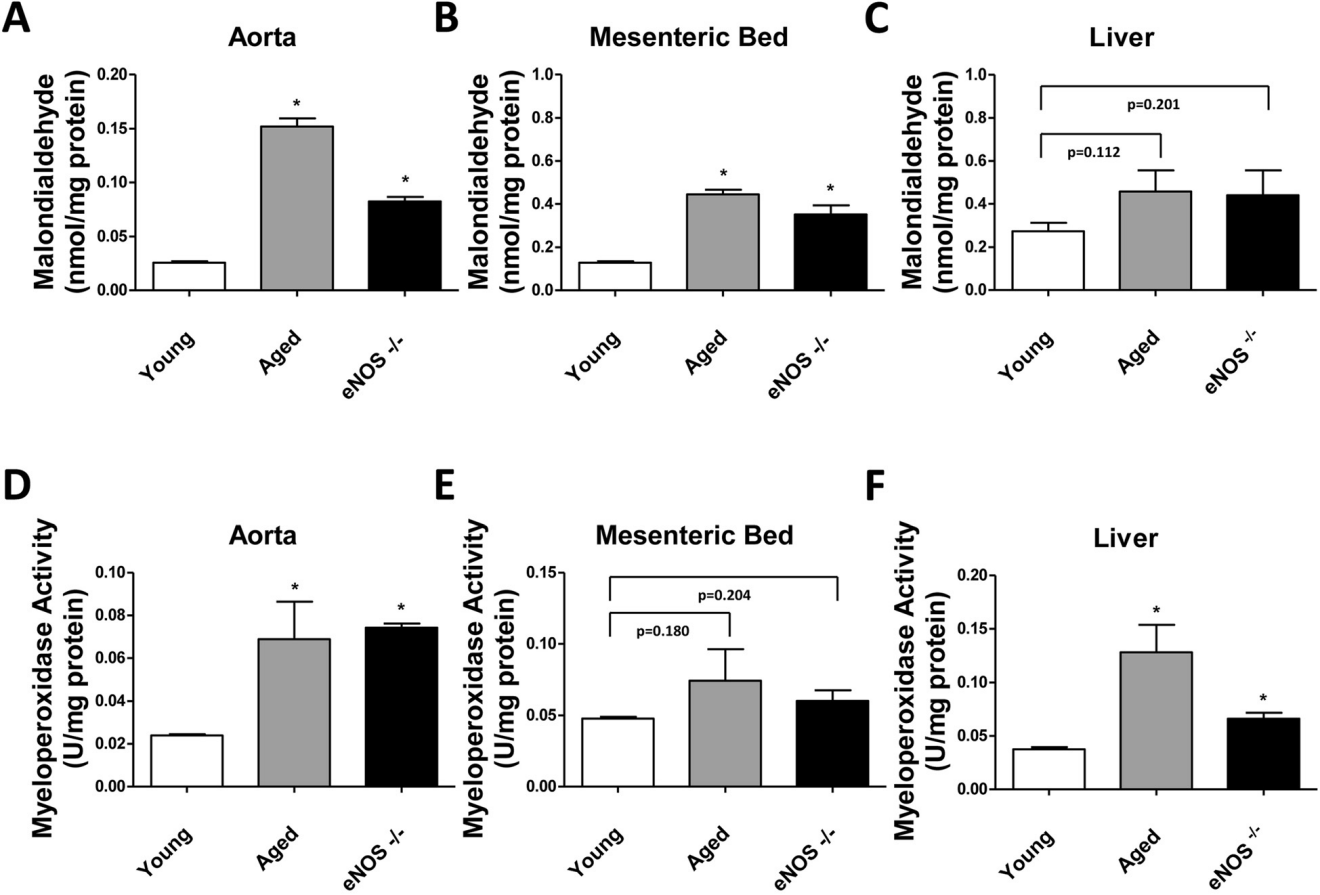
**Determination of lipid peroxidation and myeloperoxidase activity in aorta, mesenteric bed and liver harvested from CLP-injured mice.** Tissue malondialdehyde (MDA) levels (index of cellular injury/oxidative stress) and myeloperoxidase (MPO) activity (used as a marker of tissue inflammation) were detected in aorta **(Panel A and D)**, mesenteric vascular bed **(Panel B and E)** and liver **(Panel C and F)** harvested from young, aged and eNOS^−/−^ mice subjected to 12 hours of CLP-induced sepsis (^*^ *P* <0.05 vs. young; n = 5 tissue samples were analyzed in each group of mice). CLP, cecal ligation and puncture; eNOS, endothelial isoform of nitric oxide synthase.

In a different set of experiments, tissues and blood were harvested from eNOS^−/−^, aged and young mice subjected to 12 hours of CLP. A complete metabolic panel for detection of plasma markers of organ injury was performed through the VetScan Chemistry Analyzer (Table 1). The analysis revealed a slight trend of CLP-induced increase of the liver injury markers ALT and ALP and the pancreatic injury marker AMY in wild-type mice. Significantly higher increases were noted in the same parameters, both in the aged and eNOS^−/−^ mice. Blood urea nitrogen (BUN), phosphorus (PHOS) and creatinine (CRE) levels (index of renal and hepatic failure) increased in the eNOS^−/−^ and aged mice but remained in the standard range in the young wild-type septic young mice. Analysis of tissue oxidative stress by the MDA assay and tissue mononuclear cell infiltration by the MPO activity assay were carried out in aorta, mesenteric bed (a vascular district known to be exquisitely abundant in endothelium and sensitive to eNOS/NO-dependent effects on the peripheral vascular homeostasis) and liver homogenates, showed an overall increase in sepsis-induced tissue damage both by aging and by eNOS deficiency (Figure 2).

**Table 1:** **Comprehensive metabolic panel in mice subjected to CLP**

**SHAM**										**CLP**								
	**Young**			**Aged**			**eNOS−/−**			**Young**			**Aged**			**eNOS−/−**		
	**Mean**	**SEM**	**N**	**Mean**	**SEM**	**N**	**Mean**	**SEM**	**N**	**Mean**	**SEM**	**N**	**Mean**	**SEM**	**N**	**Mean**	**SEM**	**N**
ALB (g/l)	30.20	1.32	5	30.40	1.12	5	32.20	0.73	5	22.00*	4.16	5	22.00*	1.53	5	17.00*	0.58	5
ALP (U/l)	58.20	4.31	5	67.60	7.22	5	69.40	1.36	5	82.00*	13.87	5	102.33*#	25.34	5	96.00*#	2.89	5
ALT (U/l)	29.80	2.52	5	41.20	6.05	5	47.00	2.63	5	50.00*	1.73	5	77.00*#	7.51	5	89.67*#	21.67	5
AMY (U/l)	869.60	38.09	5	943.80	38.30	5	915.00	41.37	5	1117.00*	117.60	5	1346.67*#	174.79	5	907.00	201.53	5
TBIL (μM)	4.79	0.34	5	5.47	0.34	5	5.47	0.34	5	4.67	0.33	5	4.33	0.67	5	5.33	0.67	5
BUN (mM)	7.43	0.21	5	7.71	0.54	5	10.21	0.14	5	10.23	1.05	5	25.93*#	1.79	5	28.90*#	1.40	5
Ca^2+^	2.20	0.01	5	2.29	0.06	5	2.28	0.05	5	1.86	0.07	5	2.12	0.13	5	2.11	0.02	5
PHOS (mM)	1.87	0.10	5	2.32	0.09	5	2.02	0.12	5	2.66*	0.42	5	4.57*#	0.23	5	4.78*#	0.64	5
CRE (μM)	19.45	1.77	5	31.82	6.00	5	24.75	5.15	5	19.67	1.67	5	25.67*#	7.67	5	27.33*#	9.3305	
GLU (mM)	12.90	0.50	5	12.38	1.76	5	12.28	1.27	5	10.17	3.12	5	8.50	0.89	5	11.80	3.85	5
Na^+^ (mM)	141.20	0.97	5	147.00	1.64	5	144.20	1.69	5	150.00	3.51	5	149.33	1.20	5	152.33	3.53	5
K^+^ (mM)	5.34	0.23	5	6.66	0.26	5	6.00	0.48	5	5.40	0.96	5	5.80	0.25	5	8.87	0.52	5
TP (g/l)	48.80	0.80	5	49.80	2.00	5	47.80	0.70	5	35.33	3.38	5	27.63	11.87	5	35.67	1.76	5
GLOB (g/l)	18.20	1.00	5	19.60	1.40	5	14.80	0.20	5	13.67	1.67	5	21.00	5.00	5	18.67	2.33	5

Determinations of alanine aminotransferase (ALT), albumin (ALB), alkaline phosphatase (ALP), amylase (AMY) total calcium (CA^2+^), creatinine (CRE), glucose (GLU), phosphorus (PHOS), potassium (K^+^), sodium (NA^+^), total bilirubin (TBIL), total protein (TP), globulin (GLOB) and urea nitrogen (BUN) were performed by VetScan Chemistry Analyzer (Abaxis) on 100 μl of heparinized whole blood collected from young, aged and eNOS-deficient mice subjected to 12 hours of severe sepsis (CLP) or sham operation. *(*P* <0.05) vs*.* corresponding sham-operated group. ^#^(*P* <0.05) vs*.* young CLP mice (n = 5 per group). Please notice the marked degree of multiple organ dysfunctions induced by sepsis in aged and eNOS knockout mice. CLP, cecal ligation and puncture.

### eNOS deficiency and aging both enhance the degree of cardiovascular oxidative damage and the degree of endothelial dysfunction in sepsis

**Figure 3:**
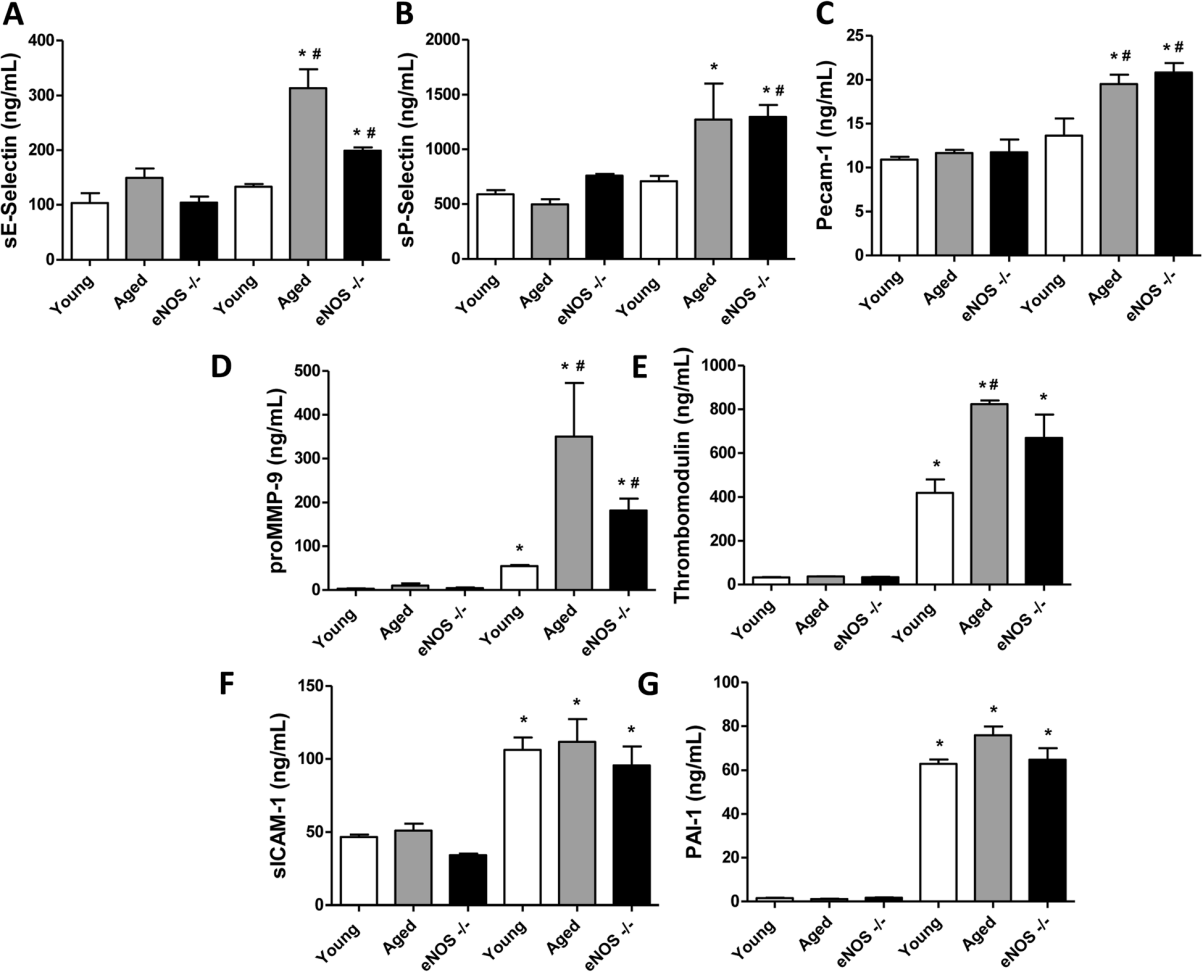
**Detection of plasma markers of cardiovascular dysfunction (CVD panel) in sham-operated or CLP-injured mice.** Plasma levels of sE-Selectin **(Panel A)**, sP-Selectin **(Panel B)**, Pecam-1 **(Panel C)**, proMMP-9 **(Panel D)**, thrombomodulin **(Panel E)**, sICAM-1 **(Panel F)**, and PAI-1 Total **(Panel G)**, were simultaneously detected in young, aged or eNOS^−/−^ mice 12 hours following sham (left three columns in each panel) or CLP (right three columns in each panel) procedure. (^*^ *P* <0.05 vs. corresponding sham-operated mice and ^#^ *P* <0.05 vs. young CLP; n = 5 individual plasma samples were tested from each group of mice). CLP, cecal ligation and puncture; eNOS, endothelial isoform of nitric oxide synthase.

**Figure 4:**
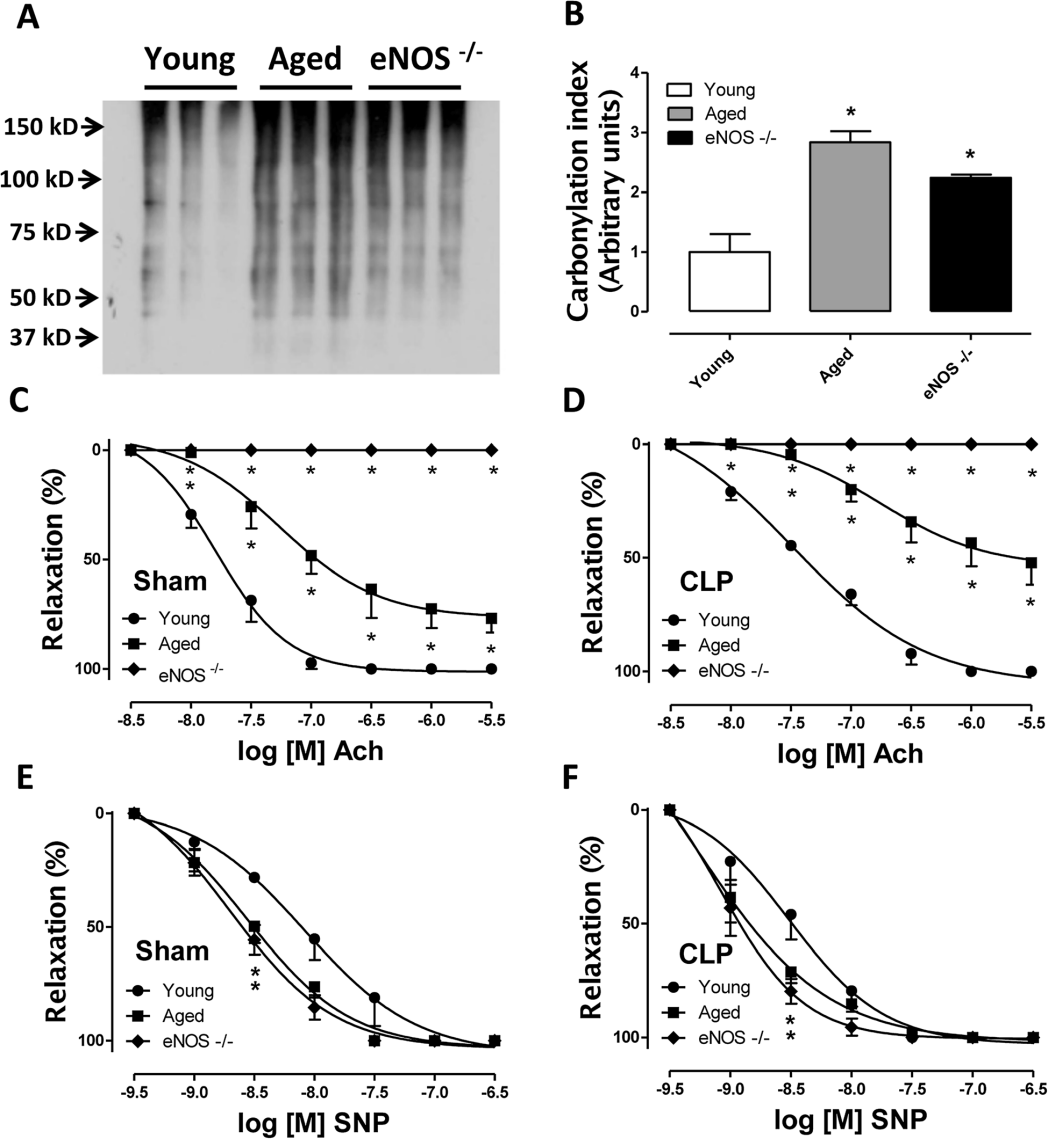
**Assessment of oxidative damage and endothelial dysfunction in aortas isolated from septic mice. (Panel A)** Following CLP, young, aged and eNOS^−/−^ mice were euthanized by opening the chest cavity under deep anesthesia and the thoracic aortas were isolated. Twenty μg of protein extracts were subjected to SDS-PAGE and derivatized protein carbonyl groups were immunodetected by OxyBlot (Merck Millipore). Immunoreactivity was visualized by chemiluminescent detection. Blots were scanned and a densitometric analysis was performed **(Panel B)** (^*^ *P* <0.05 vs. young mice; n = 3). In a separate set of experiments, aortic rings (approximately 2 mm in length) were harvested from sham-operated and CLP-injured mice, placed in 5 ml organ baths filled with oxygenated (95% O_2_ to 5% CO_2_) Krebs-Henseleit solution at 37°C and mounted onto isometric force transducers. Concentration response curves to acetylcholine (Ach) **(Panel C and D)** and sodium nitroprusside (SNP) **(Panel D and E)** were performed on a stable phenylephrine-induced tone. Please notice the inability of the aortic rings from eNOS^−/−^ mice to respond to Ach stimulation. Aged mice showed a basal level of endothelial dysfunction **(Panel C)** (^*^ *P* <0.05 vs. young). CLP induced a shift to the right of the concentration response curve to Ach, which was more pronounced in the aged mice compared to the young **(Panel D)** (mean LogEC_50_ from −7.797 to −7.488 in young mice; mean LogEC_50_ from −7.261 to −6.762 in aged mice). Increased sensitivity to the nitric oxide (NO) donor SNP was also seen in aged and eNOS-deficient vessels **(Panel E and F)**. CLP, cecal ligation and puncture; eNOS, endothelial isoform of nitric oxide synthase.

Plasma levels of sE-Selectin, sICAM-1, Pecam-1, sP-Selectin, PAI-1 total, proMMP-9, and thrombomodulin were simultaneously detected in young, aged or eNOS^−/−^ mice 12 hours following CLP or sham procedure (Figure 3). Low baseline values of the markers of endothelial dysfunction were detected in sham-operated mice, grossly unchanged within the groups. Although 12 hours is a relatively short time to expect increased plasma levels of sE-Selectin, sP-Selectin and Pecam-1 in septic young mice compared to the sham group, their levels did increase significantly in the aged and eNOS-deficient septic mice. The analysis of the data revealed a similar trend of increase for pro-MMP-9 and thrombomodulin. Twelve hours of CLP induced about 15-fold increase in both pro-MMP-9 and thrombomodulin plasma levels in young septic mice compared to young, sham-operated mice. More pronounced increases were detected in the septic eNOS^−/−^ with the septic aged mice showing the highest levels among the groups. No statistically significant differences were detected between young, aged and eNOS-deficient septic mice for plasma levels of SICAM-1 and PAI-1. Detection of protein carbonyl groups, index of oxidative damage, was performed by OxyBlot analysis of aortae isolated from mice subjected to CLP. As shown in Figure 4A and B, some protein carbonylation was already detectable in wild-type septic mice, but the induction (measured by software-assisted densitometric analysis of the chemiluminescent signal) increased more substantially (by about three- and two-fold, respectively) in aged mice and eNOS-deficient mice. Dysfunction of the endothelium during sepsis was determined by assessing the Ach-induced vasorelaxant responses *in vitro* in aortic rings precontracted with phenylephrine. As expected, aortic rings from eNOS^−/−^ mice were completely unable to relax in response to Ach (Figure 4C and D). Aged mice showed some basal level of endothelial dysfunction, as evidenced by a shift to the right of the sigmoidal concentration response curve to Ach in the sham group. CLP-induced sepsis produced a significant degree of endothelial dysfunction, the severity of which was more profound in the aged mice compared to the young mice (mean LogEC_50_ from −7.8 to −7.5 in young mice, sham versus CLP; mean LogEC_50_ from −7.3 to −6.8 in aged mice, sham versus CLP). Moreover, both aged and eNOS-deficient vessels showed increased sensitivity to the NO donor SNP (Figure 4E and F).

### Severity of endothelial dysfunction, a predictor of mortality in mice during sepsis

**Figure 5:**
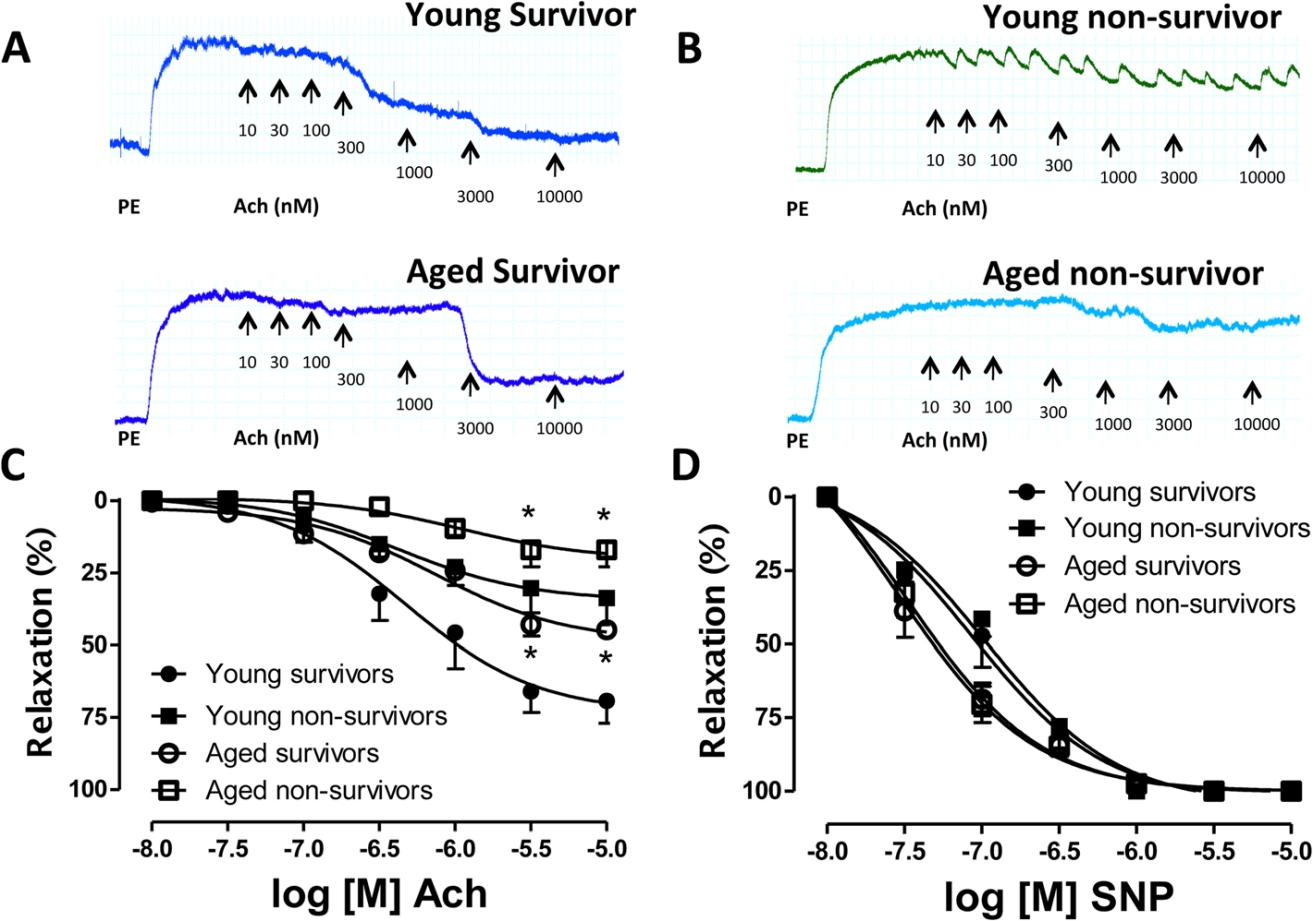
**Assessment of endothelial function in mice that survived or did not survive 48 hours of sepsis.** CLP was performed in C57BL/6 young mice as described in Methods. Following CLP, mice were constantly monitored for 48 hours. In our model the survival rate at 48 hours is approximately 50%. Thoracic aortas from the mice that did not survive the 48 hours of sepsis were harvested at the exact time of death, placed in 5 ml organ baths filled with oxygenated Krebs-Henseleit solution at 37°C and mounted onto isometric force transducers to assess for vascular response to endothelium-derived nitric oxide (NO) by acetylcholine (Ach) stimulation. Five death events were recorded, aortic rings from the five non-survivors were used. The mean time of survival (or tissue harvesting) was 39 hours. Survivors were euthanized at 48 hours of sepsis by opening the chest cavity. In a sequent study, a similar approach was used for the assessment and comparison of endothelial function in survivor versus non-survivor aged mice. Since the survival rate at 48 hours of CLP is only 80% in the case of the aged mice, we restricted the CLP to a 24-hour time frame. Aortic rings were harvested from five different mice. The mean time of survival (or tissue harvesting) in this group was 17 hours. The survivors were euthanized 24 hours following CLP. Please note the marked degree of endothelial dysfunction in the non-survivor group of animals **(Panel A, B and C)**. Concentration response curves to the NO donor sodium nitroprusside (SNP) were performed to ensure the integrity of the smooth muscle and the viability of the tissue preparations **(Panel D)**. (^*^ *P* <0.05 vs. corresponding survivors). CLP, cecal ligation and puncture.

In the current model of severe sepsis, the survival rate in the young group of mice was approximately 50% at 48 hours. In the next set of studies, we aimed to investigate whether the degree of endothelial dysfunction correlates with the outcome of sepsis. Mice were divided into two groups: those that survived and those that did not survive 48 hours of sepsis. Mice were closely monitored for the 48-hour period of time and thoracic aortae from the non-survivors were harvested at the time of death and processed for assessment of *in vitro* vascular response to endothelium-derived NO by Ach stimulation. Mice that survived the 48 hours of sepsis were euthanized by opening the chest cavity and the aortic ring assay was performed subsequently. A distinct difference of severity of endothelial dysfunction was detected between the young mice that survived and those that died (Figure 5). In a subsequent study, a similar approach was used for the assessment and comparison of endothelial function in survivor versus non-survivor aged mice. Since the survival rate at 48 hours of CLP is only 80% in the case of the aged mice, we restricted the CLP to 24 hours. Aortic rings were harvested from five non-survivor mice at the time the death was scored (see figure legend for additional methodological details). The survivors were euthanized 24 hours following CLP. These data indicate that endothelial dysfunction precedes mortality and suggest that it may be a predictor of it.

### eNOS deficiency and aging both produce a worsening of sepsis-induced mitochondrial dysfunction in sepsis

**Figure 6:**
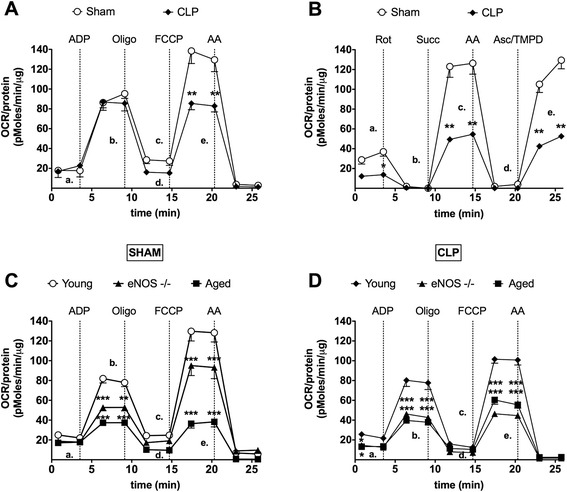
**Bioenergetic analysis in isolated liver mitochondria from mice subjected to sham or CLP operation.** Mitochondrial respiration/function was determined by oxygen consumption rate (OCR) utilizing the Seahorse XF24 Extracellular Flux Analyzer (Seahorse Bioscience). During the coupling experiment in isolated mitochondria **(A, C, D)**, the assay media contains succinate, as a complex II substrate and rotenone as a complex I inhibitor. Sequential measurement of basal, State 2 **(a)**, State 3 **(b)**, State 4o **(c)** and the uncoupler-stimulated respiration, State 3u **(d)** were performed through the sequential injections of ADP, oligomycin, FCCP and antimycin. Please note that the OCR after the addition of FCCP (State 3u) is much higher in sham group **(A)**. Furthermore, a significant impairment in State 3 and State 3u respirations has been detected in aged and eNOS^−/−^ group compared to young mice subjected to CLP intervention or the sham group **(C, D). (Panel A**, **C**, **D**, ^**^ *P* <0.01 and ^***^ *P* <0.001 vs. sham or CLP-operated young mice; n = at least 4/group). The experiment in **(panel B)** demonstrates the electron flow activity through the different complexes of the electron transport chain. The assay media contains pyruvate/malate as substrates of complex I, and FCCP to uncouple the mitochondrial function. In uncoupled mitochondria all complexes can be examined individually by the sequential injection of rotenone, succinate, antimycin and TMPD/ascorbate. All these mitochondrial toxins and substrates permit to observe 1., the function of complex I **(a)**, 2., the inhibition of complex I **(b)**, 3., the function of complex II **(c)**, 4., the inhibition of complex II **(d)**, 5., the activity of cytochrome C/complex IV **(e)**. Overall, all mitochondrial complexes show better function in mitochondria isolated from the liver of the sham group compared to the other groups **(Panel B**, ^**^ *P* <0.01 vs. sham-operated mice; n = at least 4/group). ADP, adenosine 5′-diphosphate sodium salt; CLP, cecal ligation and puncture; eNOS, endothelial isoform of nitric oxide synthase; FCCP, carbonylcyanide-4-trifluorometh-oxyphenylhydrazone; TMPD, tetra-methyl-p-phenylenediamine dihydrochloride.

CLP caused a significant reduction of the total mitochondrial function based on different bioenergetic measurements (‘coupling’ and ‘electron flow’ assays) in isolated mouse liver mitochondria *ex vivo* (Figure 6A and B), indicative of an overall, sepsis-associated impairment of mitochondrial function. A decrease in oxygen consumption rate (OCR) was also noted both in sham-operated aged and eNOS^−/−^ mice compared to the sham-operated young mice (all in the absence of CLP). CLP, in turn, tended to further worsen mitochondrial function in all three groups (Figure 6C and D).

## Discussion

The vasculature, under normal conditions, produces local endothelium-dependent vasodilatory mediators, the most important of which is nitric oxide (NO). The loss of endothelial function in sepsis is known to promote impaired local regulation of vascular responsiveness; impaired vascular autoregulation, tissue metabolism/perfusion mismatch, increased intravascular coagulation, and platelet/neutrophil adhesion/activation [8,20–22]. One of the principal causes of mortality in sepsis is progressive vascular dysfunction. As first shown over 20 years ago [3,4], and repeatedly confirmed by multiple studies since then [5–14], various forms of circulatory shock are associated with the development of endothelial dysfunction, that is an impaired ability of the endothelial cells to produce NO. Several lines of evidence support the view that endothelial dysfunction is a key factor in promoting coagulation abnormalities, multiple organ failure and mortality in sepsis in humans, as well [9,21]. Importantly, in septic patients, the degree of the loss of endothelium-dependent vasorelaxant responsiveness shows a close correlation with mortality [13,14,23].

Endothelial dysfunction promotes neutrophil adhesion and infiltration into tissues, which is a key contributor to MODS [21–27]. In addition, endothelial dysfunction in sepsis leads to perfusion/oxygenation mismatch and coagulation abnormalities, culminating in mortality in sepsis [8,20,21]. Supplementation of NO has been shown to improve the outcome of various forms of shock [24–27]. These findings support the hypothesis that impairment of endothelium-dependent relaxations is an important pathogenetic process in sepsis. Consequently, it has been previously suggested [21–28] that therapeutic approaches that preserve endothelial function have the potential to improve sepsis outcomes.

Even though sepsis is a disease of the aged population, it is surprising that, as of to date, all of the published studies investigating endothelial dysfunction in sepsis have used neonatal or young/adult animals. In other words, the molecular mechanisms of endothelial dysfunction in sepsis have never been specifically explored in aged animals, and all of the currently published reports on the pathogenesis of endothelial dysfunction in sepsis are incomplete, as they do not take into account this key aspect of the disease. The results of the present study show that endothelial dysfunction (evidenced both by functional studies, that is endothelium-dependent relaxation, as well as by measuring multiple plasma indices of endothelial cell dysfunction) is early-onset and particularly severe in aged mice subjected to CLP. Previous studies suggest that aging is associated with impaired bioavailability of NO, at least in part, due to a downregulation of endothelial eNOS expression and activity [29,30] and that mice with genetic eNOS deficiency exhibit an accelerated cardiovascular aging phenotype [31]. The present results demonstrate that - similar to aging [32], eNOS deficiency also exacerbates MODS and mortality in the currently used rodent model of sepsis. In addition, both eNOS-deficient and aged mice exhibit a higher degree of sepsis-associated multiple organ dysfunction, tissue inflammation and oxidative stress. Moreover, both eNOS-deficient and aged mice exhibit a high degree of vascular oxidative damage in CLP-induced sepsis. In addition, there is a significant worsening of sepsis-induced mitochondrial dysfunction, both in eNOS-deficient mice and in aged mice. Comparison of the surviving and non-surviving groups of animals indicates that the severity of endothelial dysfunction may be a predictor of mortality of mice subjected to CLP-induced sepsis. Taken together, the current results suggest (but do not directly prove) that endothelial dysfunction may be an independent contributor as well as early predictor of MODS and mortality in sepsis.

It is worth mentioning that a recent study by Raeven and colleagues has used a similar approach to our study in the sense that it compared endothelial dysfunction marker levels (PAI-1, thrombomodulin) in dying/surviving subgroups of animals subjected to CLP (in this study, the context was to determine if a pre-existing trauma amplifies subsequent CLP outcomes). In this study, significantly higher PAI-1 levels were found in the non-surviving septic mice as opposed to the survivor groups, while no differences were noted with respect to thrombomodulin levels [33]. This study also concluded that most of the PAI-1 release is not the consequence of endothelial dysfunction, but is derived from other sources (such as the liver). Interestingly, in the current study, PAI-1 was not a marker that was exacerbated by either eNOS deficiency or by aging in response to CLP (Figure 3) (as opposed to many other markers of endothelial dysfunction that were exacerbated, including thrombomodulin). When taken together, these studies with the current report, we may conclude that pre-existing aging or eNOS deficiency may exacerbate the outcome of CLP via mechanisms that may (at least in part) depend on the development of endothelial dysfunction, while the amplifying effect of pre-existing trauma mainly involves other mechanisms.

What, then, may be the potential molecular mechanisms underlying the development of endothelial dysfunction in aging animals subjected to sepsis? First of all, there is already a 'baseline' level of endothelial dysfunction in the aging organism. This has been well documented, both in the absence and in the presence of additional risk factors (for example hypertension, diabetes, smoking) [34–38]. The causes of this dysfunction are at least four-fold. The first factor is oxidative stress. Increased production of reactive oxygen species ((ROS); from mitochondrial sources as well as due to an increased activity of NAD(P)H oxidases) is prevalent in aged blood vessels [34–40]. The impairment of NO bioavailability in aging is, in part, due to direct inactivation of NO by superoxide, but this response is further aggravated by an age-related decline in eNOS expression, reduced availability of tetrahydrobiopterin and/or a decreased intracellular L-arginine [34–40]. The second factor is an enhanced activation of pathways downstream of oxidative stress. Many of the adverse consequences of oxidative stress are mediated via production of peroxynitrite: a substantially enhanced cardiovascular peroxynitrite formation is present in aged blood vessels. Downstream targets of peroxynitrite-induced cytotoxicity are multiple and include vascular poly (ADP-ribose) polymerase (PARP) overactivation [41–44]. The third factor is a depressed activity/expression of antioxidant enzymes. The pathomechanisms of vascular aging are intimately linked to reduced antioxidant defenses, including inhibition of various antioxidant enzymes (for example superoxide dismutase), as well as reduced levels of antioxidant factors (for example glutathione). A central and specific component of this attenuated antioxidant response is the inability of the aged cells to activate Nrf2, a key master switch of antioxidant defense in the cell [45–50]. Finally, the fourth factor is a low-grade vascular inflammation. In aged blood vessels, reactive oxygen/nitrogen species (ROS/RNS)-dependent signaling induces proinflammatory responses, which, then, form a positive-feedback circle by further promoting cellular oxidative stress (for example by activation of NAD(P)H oxidases) [51–53].

Since circulatory shock is known to activate some of the same pathways of vascular injury as in aging, including oxidative stress, downstream pathways of oxidant injury, suppression of antioxidant defenses and vascular (as well as systemic) inflammation [54–61], it is not entirely surprising that in aged blood vessels, circulatory shock induces an particularly severe form of endothelial dysfunction, in which oxidative/nitrosative processes become a dominant cause of the injury. Based on recent data in various *in vitro* and *in vivo* experimental systems [49,50] we hypothesize that this phenomenon is, to a significant degree, due to the fact that aged blood vessels lose their ability to activate Nrf2, and thereby they are unable to mount proper antioxidant defenses, leading to an increased oxidative-nitrosative stress, and hyperactivation of downstream damage pathways. This, however, remains to be further investigated in future studies.

Endothelium-derived NO plays a key role in regulation of mitochondrial function and cellular energy balance [62–64]. On the basis of the aforementioned studies, we also hypothesize that the exacerbation of ROS/RNS-mediated responses and endothelial dysfunction are key contributors to the development of mitochondrial dysfunction demonstrated in the current study. According to multiple preclinical and clinical studies, mitochondrial dysfunction (or 'cytopathic hypoxia') is a significant contributor to organ dysfunction and mortality in various forms of critical illness [65–67]; based on the current data, we hypothesize that a suppression of endothelial NO production may be one of the upstream/proximate causes of this defect. It was also interesting to note that eNOS deficiency, as well as aging, both produced a comparable degree of 'baseline' suppression of mitochondrial function. Although the mechanisms responsible for these findings remain to be further explored, we hypothesize that suboptimal levels of tissue NO may lead to an exacerbation of oxidative stress, which, in turn, may damage mitochondrial electron transport proteins, and may contribute to a dysfunction of the mitochondrial electron transport chain.

We realize that the current study has a number of limitations. We have conducted, on one hand, a comparison of normal young mice subjected to CLP with old mice subjected to CLP. On the other hand, we compared septic (young) eNOS knockouts subjected to CLP to the young wild-type mice subjected to CLP. The similarities of the exacerbation of the response to CLP are striking, but we admit that these similarities and parallels only suggest (but do not prove) causality. One future experiment to get closer to probing causality may consist of the induction of CLP in aged eNOS-deficient mice; another future experiment may consist of the therapeutic/pharmacological replacement of NO in aged mice subjected to CLP. We must also point out that the current experimental design did not use antibiotics during CLP. In the field of CLP, some investigators routinely use antibiotics and others do not. It appears to us that many (but not all) investigators who are interested in basic pathological processes first start out with CLP models without antibiotics, while many (but not all) investigators who are more focused on therapeutic/translational approaches tend to use antibiotics. As the current study was more focused on basic pathophysiological mechanisms and not (yet) therapeutic interventions, we selected a model without antibiotics for the initial set of experiments. However, future follow-up studies may incorporate antibiotics as well. Finally, additional studies in outbred mice (as opposed to inbred mice) and comparison of the results in male and female mice will be necessary to probe and potentially extend the validity of the current findings.

## Conclusions

**Figure 7:**
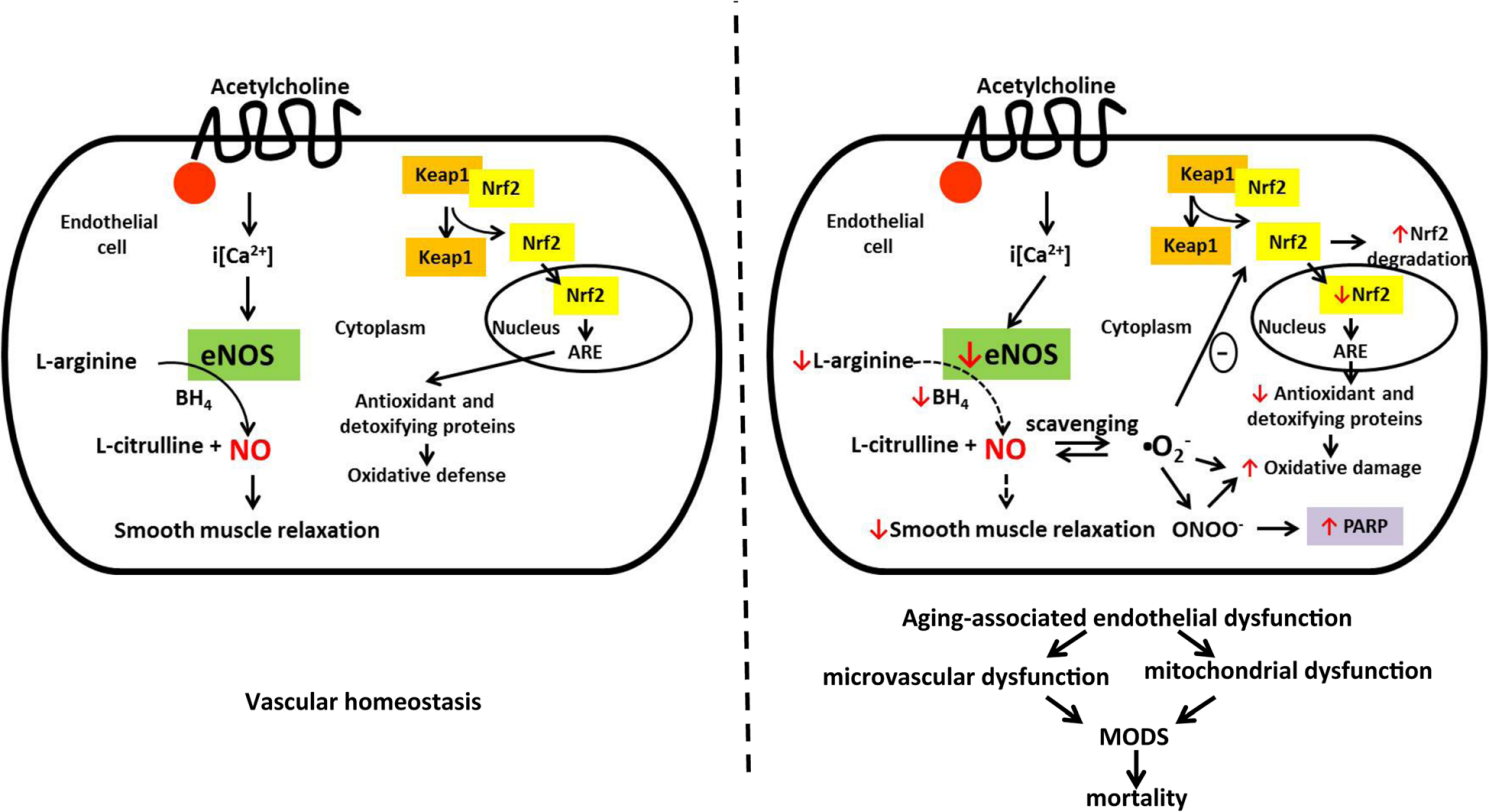
**Potential pathways and mechanisms underlying the enhancement of endothelial dysfunction in aged mice subjected to sepsis.** In aged blood vessels, septic shock induces an overwhelming degree of oxidative damage, leading to a marked degree of endothelial dysfunction, which, in turn, contributes to the MODS and mortality of sepsis. We hypothesize that part of the process involves the fact that in aged endothelial cell, oxidative stress is unable to induce an appropriate activation of the Nrf2/ARE pathway. Please see Discussion for additional details. ARE, antioxidant response element; BH4, tetrahydrobiopterin, a co-factor of eNOS; eNOS, endothelial isoform of NO synthase; iCa^2+^, intracellular calcium concentration; Keap1, Kelch-like erythroid cell-derived protein with CNC homology (ECH)-associated protein 1; MODS, multiple organ dysfunction syndrome; NO, nitric oxide; Nrf2, nuclear factor erythroid 2 (NF-E2)-related factor 2; PARP, poly(ADP-ribose) polymerase.

Taken together, based on the results of the current study, and based on published reports in the literature, we propose the following working model (Figure 7) of pathophysiological events in aged animals subjected to sepsis. (1) In the aging organism, there is a baseline endothelial dysfunction, which is the consequence of increased oxidative stress and low-level vascular inflammation, all of which is the consequence of 'normal' or 'physiological' aging. (2) As part of the same processes, antioxidant defenses (for example Nrf2 mobilization) are weakened. (3) When subjected to systemic inflammation/sepsis, the aging organism is unable to mount an appropriate antioxidant defensive response. (4) At the same time, the sepsis-induced endothelial dysfunction and the baseline-level endothelial dysfunction, in an additive/synergistic fashion, lead to a particularly severe form of endothelial dysfunction (that is vascular NO levels are markedly suppressed). (5) The overwhelming amount of reactive oxidants, coupled with the low amount of antioxidant NO, leads to a 'tipping of the balance' of the ROS/RNS status, leading to an increased degree of oxidative protein modifications, culminating in further endothelial dysfunction, mitochondrial injury, cellular energetic impairment, cell death, culminating in a severe degree of multiple organ dysfunction and high mortality. These processes occur, at least in part, through the activation of various positive feedback cycles of cell injury. One of these cycles may involve an enhancement of pro-coagulation pathways, which are known to be markedly exacerbated in aging animals subjected to sepsis [68] and may be downstream from endothelial dysfunction.

While we realize that the current model does not consider all known pathophysiological processes of septic shock (importantly, it does not fully consider inflammatory/immune processes that are different in various stages of the disease), the focus on cardiovascular events may be justified, as prior studies have demonstrated that cardiovascular failure is the primary cause of mortality in the current model [69]. Therefore, our working hypothesis may represent a useful 'working model', the experimental probing of which will require multiple lines of additional studies. The current working model entails potential therapeutic/interventional strategies, including pharmacological approaches such as restoration of vascular NO homeostasis, restoration of antioxidant defense systems such as Nrf2 and various antioxidant/endothelial protective/mitochondrial protective strategies.

## Key messages

- eNOS deficiency and aging both exacerbated the severity of sepsis.
- This is evidenced by increased oxidative stress, MODS, and mortality.
- Vascular dysfunction is particularly severe in aged animals subjected to sepsis, and precedes and predicts mortality.
- We conclude that the lack of endothelial NO production, on its own, is sufficient to markedly exacerbate the severity of septic shock. Aging markedly worsens the degree of endothelial dysfunction in sepsis, yielding a significant worsening of the overall outcome.

## Abbreviations

Ach: acetylcholine
ADP: adenosine 5′-diphosphate sodium salt
ALB: albumin
ALT: alanine aminotransferase
ALP: alkaline phosphatase
AMY: amylase
ARE: antioxidant response element
BUN: blood urea nitrogen
Ca^2+^: total calcium
CLP: cecal ligation and puncture
CRE: creatinine
DNP: dinitrophenylhydrazone
DNPH: 2, 4-dinitrophenylhydrazine
eNOS: endothelial isoform of nitric oxide synthase
ETC: electron transport chain
FCCP: carbonylcyanide-4-trifluorometh-oxyphenylhydrazone
GLU: glucose
ICU: intensive care unit
K^+^: potassium
Keap1: Kelch-like erythroid cell-derived protein with CNC homology (ECH)-associated protein 1
MDA: malondialdehyde
MODS: multiple organ dysfunction syndrome
MPO: myeloperoxidase
Na^+^: sodium
NO: nitric oxide
Nrf2: nuclear factor erythroid 2 (NF-E2)-related factor 2
OCR: oxygen consumption rate
OXPHOS: oxidative phosphorylation
PARP: poly(ADP-ribose) polymerase
PE: streptavidin-phycoerythrin
PHOS: phosphorus
RNS: reactive nitrogen species
ROS: reactive oxygen species
SNP: sodium nitroprusside
TBIL: total bilirubin
TP: total protein
